# Back to basics: the coagulation pathway

**DOI:** 10.1007/s44313-024-00040-8

**Published:** 2024-10-28

**Authors:** Seonyang Park, Joo Kyung Park

**Affiliations:** 1 Department of Internal Medicine, Inje University Haeundae Paik Hospital, 875 Haeundae-Ro, Haeundae-Gu, Busan, 48108 Korea; 2 Daisy Hill Hospital, 5 Hospital Road, Newry, BT35 8DR UK

**Keywords:** Coagulation, Coagulation pathway, Hemostasis

## Abstract

The classic coagulation cascade model of intrinsic and extrinsic coagulation pathways, i.e. contact activation pathway and tissue factor pathway, has been widely modified. The cascade can be categorized as follows: 1) initiation by tissue factor (TF), 2) amplification by the intrinsic tenase complex, and 3) propagation on activated platelets. TF-FVIIa forms an extrinsic tenase complex and activates FX to FXa and FIX to FIXa. FXa-FVa forms a prothrombinase complex that converts prothrombin into thrombin. At this initial stage of coagulation, only small amounts of thrombin are generated owing to the low circulating levels of FVa. The generated thrombin, although in minor quantities, is sufficient to prime the subsequent coagulation reactions. Platelets and in turn FV, FVIII, and FXI are activated. Subsequently, FVIIIa binds to FIXa to form the intrinsic tenase complex, which is aided by a cofactor, FVIIIa, and activates FX at a rate 50-times higher than that of the extrinsic tenase complex, thereby amplifying thrombin generation. Thrombin cleaves fibrinogen into one fibrin monomer and two fibrinopeptides. Fibrin monomers aggregate, crosslink, and branch into an insoluble fibrin network structure. The contact activation system is initiated by FXII, which is activated upon exposure to negatively charged surfaces. Coagulation is driven by FXIIa-mediated FXI cleavage. FXIa activates FIX, which forms an intrinsic tenase complex, eventually leading to thrombin formation. The contact activation system is considered to contribute to thrombosis but is not required for hemostasis in vivo.

## Introduction

Hemostasis is a physiological process that controls bleeding when the blood vasculature is injured. It involves an intricate balance between the coagulation and fibrinolytic systems as well as platelets and endothelial cells to minimize bleeding while simultaneously preventing excessive thrombus formation and dissolving the clot after complete tissue repair. The first medical description of hemostasis dates back over a thousand years ago to Abu Qasim Khalaf Ibn Abbas Al Zahrawi (936–1013), also known as Albucasis, a pioneering surgeon who applied cauterization in multiple operations [1]. Going back to basics, this article aims to provide a brief review on the current essential features of blood coagulation and the coagulation pathway.

## Coagulation factors

Until the end of World War II, only four coagulation components were recognized: fibrinogen, prothrombin, thromboplastin (tissue factor), and calcium. The discovery of coagulation factors V (FV) and VII (FVII) followed in 1947 [2, 3] and 1950 [4], respectively.

### International nomenclature

Most blood coagulation factors are deficient in patients with hereditary coagulation disorders. They were initially named after the first patient or based on their function or origin. Some were given different names although they were later demonstrated to be the same factor. To avoid confusion, an international committee on the nomenclature of clotting factors proposed official names with designated Roman numerals in the order of their discovery [5]. However, the original names of some factors such as fibrinogen (Factor I), prothrombin (Factor II), and tissue factor (Factor III) are still commonly used in practice. Factor IV and VI were later revealed to be calcium and activated FV, respectively [Tab. 1].

**Table 1:** Nomenclature of coagulation factors

Name	Synonym
Factor I	fibrinogen
Factor II	prothrombin
Factor III	tissue factor
Factor IV	calcium
Factor V	proaccelerin, labile factor
Factor VI	old name of Factor Va
Factor VII	proconvertin, stable factor
Factor VIII	antihemophilic factor A
Factor IX	antihemophilic factor B
Factor X	Stuart-Prower factor
Factor XI	plasma thromboplastin antecedent
Factor XII	Hagemen factor
Factor XIII	fibrin-stabilizing factor

Many coagulation factors exist as inactive zymogens that are converted into active proteolytic enzymes after limited proteolysis, whereas tissue factor (TF), FV, and FVIII function as cofactors. Cofactors enhance the action of proteolytic coagulation factors on TF-bearing cells such as endothelial cells and platelets. Fibrinogen is a structural protein, whereas factor XIII (FXIII, fibrin-stabilizing factor) is a transglutaminase.

### Vitamin K-dependent coagulation factors

Vitamin K-dependent coagulation factors require vitamin K to perform their functions. These include prothrombin, FVII, factor IX (FIX), and factor X (FX). Natural anticoagulants, such as proteins C and S, are also vitamin K-dependent factors. Vitamin K-dependent coagulation factors possess unique molecular structures and mechanisms of action. They share a similar structure of a C-terminal serine protease domain and an N-terminal γ-carboxy glutamic acid (Gla) domain, a domain characteristic of the vitamin K-dependent proteins. They are connected by epidermal growth factor (EGF)-like or kringle-like domains. Each domain has important functions, such as substrate recognition, cofactor interaction, and binding to a negatively charged lipid surface on activated platelets or endothelial cells [6].

Glutamic acid residues are converted to carboxyglutamic acid residues by a specific γ-carboxylase. This reaction requires oxygenation, carbon dioxide, and reduced vitamin K in the form of hydroquinone. Carbon dioxide is incorporated onto the γ-carbon providing a second carboxylate group on that residue. In this process, the reduced vitamin K is converted into an epoxide. Reduced vitamin K is recycled by a specific epoxide reductase, a reaction that can be blocked by warfarin and its analogues [6].

## Coagulation pathways

The concept of the intrinsic and extrinsic coagulation pathways was introduced in 1955 and cited in the literature in 1957 [7]. Both intrinsic (contact activation) and extrinsic (tissue factor) pathways trigger blood coagulation. A waterfall cascade scheme has been proposed to describe the sequential activation of coagulation factors leading to insoluble fibrin formation [8, 9]. Multistep coagulation in higher vertebrates may represent an evolutionary effort to ensure more effective clotting and prompt healing of injured vessels.

### Current concept of the coagulation scheme: tissue factor pathway initiation

Previously, TF-FVIIa was shown to initiate the extrinsic pathway (TF pathway) by activating X to form Xa, followed by the conversion of prothrombin to thrombin. In 1977, it was further discovered that TF-FVIIa activates FIX to form FIXa, thereby triggering the intrinsic coagulation pathway [10]. Although the physiological importance of these findings has not been well recognized for a long time, these observations, together with the accumulated knowledge of the biochemistry of hemophilias, form the basis of our current understanding of the blood coagulation process [11].

The coagulation process can be categorized as follows: 1) initiation by TF, 2) amplification by the intrinsic tenase complex, and 3) propagation on activated platelets [12–14]. Although the contact activation system initiated by FXIIa can contribute to thrombosis, the general consensus is that it is not required for hemostasis [15].

**Fig. 1:**
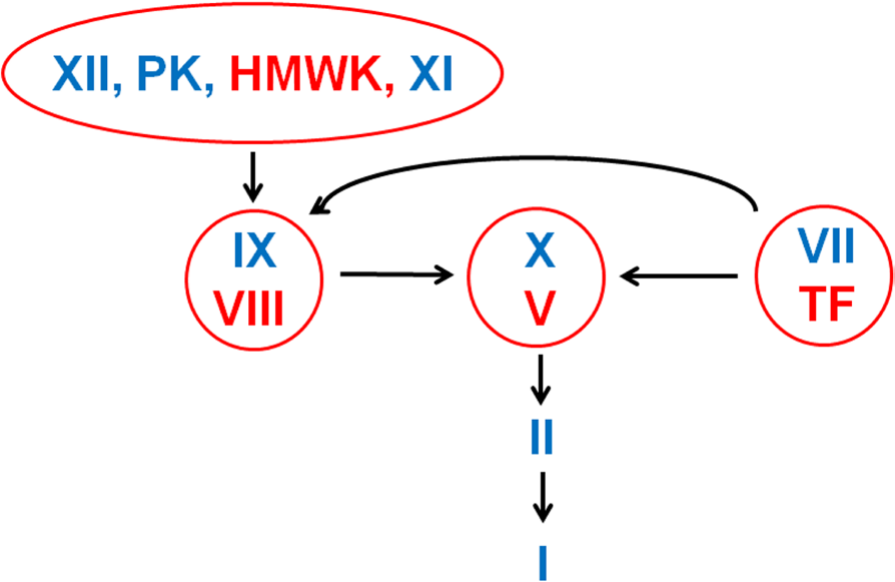
Basic concept of blood coagulation. TF-FVIIa forms the extrinsic tenase complex and activates FIX to FIXa as well as FX to FXa. FXa-FVa forms the prothrombinase complex and converts prothrombin to thrombin. At this initiation stage of coagulation, only small amounts of thrombin are generated due to the low circulating levels of FVa. The generated thrombin, although in minor quantities, is sufficient to prime the subsequent coagulation reactions. The platelets as well as FV, FVIII, and FXI are activated. Subsequently, FVIIIa binds FIXa to form the intrinsic tenase complex, which activates FX at a 50-fold higher rate than that by the extrinsic tenase complex, thereby amplifying thrombin generation. Thrombin then cleaves fibrinogen into a fibrin monomer and two fibrinopeptides. Fibrin monomers aggregate, cross-link, and branch into an insoluble fibrin network structure. The intrinsic, contact activation system is initiated by FXII, which is activated when exposed to negatively charged surfaces. The initial step involves activation of plasma prekallikrein to kallikrein, and kallikrein reciprocally activates additional FXII molecules. Coagulation is driven by FXIIa-mediated cleavage of FXI. FXIa activates FIX, which can form intrinsic tenase complex with FVIIIa, eventually leading to thrombin formation. While the contact activation system contributes to thrombosis, it is not required for hemostasis

Recently, He et al. described the concept of a coagulation scheme using in vitro coagulation assay data with various coagulation-triggering agents [14]. The basic concept of blood coagulation is illustrated in Fig. 1. TF is constitutively expressed in most cells but is not normally exposed to blood flow. It may be exposed to blood flow when the vasculature is injured. Thereafter, TF acts as a clotting trigger to initiate the coagulation cascade. TF bind to FVII to form an extrinsic tenase complex, within which FVII is activated to FVIIa. The extrinsic tenase complex converts FIX to FIXa and FX to FXa.

FXa binds to its cofactor FVa to form the prothrombinase complex. Prothrombin is converted to thrombin after proteolytic cleavage to produce prothrombin fragment 1 + 2. At this initial stage of coagulation, only small amounts of thrombin are generated owing to the low circulating levels of FVa.

Thrombin, although present in minor quantities, primes subsequent coagulation reactions to achieve active and efficient thrombin formation. It can activate platelets, as well as FV and FVIII to FVa and FVIIIa to form cofactors for Xa and IXa, respectively. It also activates FXI to FXIa, further reinforcing FIX activation.

FIXa generated on TF-bearing cells is only slowly inhibited by plasma inhibitors and can therefore make its way to the primed platelet surface. FVIIIa binds to FIXa to form an intrinsic tenase complex that activates FX at a rate that is 50-times higher than that of the extrinsic tenase complex, thereby amplifying thrombin generation. This phenomenon is referred to as a thrombin burst. Proteinase complexes that activate zymogen precursors require calcium and phospholipids.

Thrombin then cleaves fibrinogen into a fibrin monomer and two fibrinopeptides, fibrinopeptide A and fibrinopeptide B. Fibrin monomers aggregate, cross-link, and branch into an insoluble fibrin network structure. Crosslinking is dependent on the activation of FXIII, a pro-transglutaminase activated by thrombin [14].

### The contact activation system

The contact activation system, also known as the intrinsic coagulation system, is initiated by FXII. FXII is activated to FXIIa when in contact with anionic surfaces, such as subendothelial collagen in vivo or in vitro. It can activate multiple pro-inflammatory pathways as well as the intrinsic coagulation pathway. The initial step involves the conversion of plasma prekallikrein to kallikrein, which reciprocally activates additional FXII molecules.

Coagulation is driven by FXIIa-mediated FXI cleavage. FXIa activates FIX, which forms an intrinsic tenase complex with the cofactor FVIIIa, eventually leading to thrombin formation and clotting. Although the contact activation system contributes to thrombosis, it is not required for hemostasis. The contact activation system crosstalks with multiple physiological defense mechanisms, including coagulation, inflammation, fibrinolysis, and the complement system [15].

## Regulation of coagulation

**Fig. 2:**
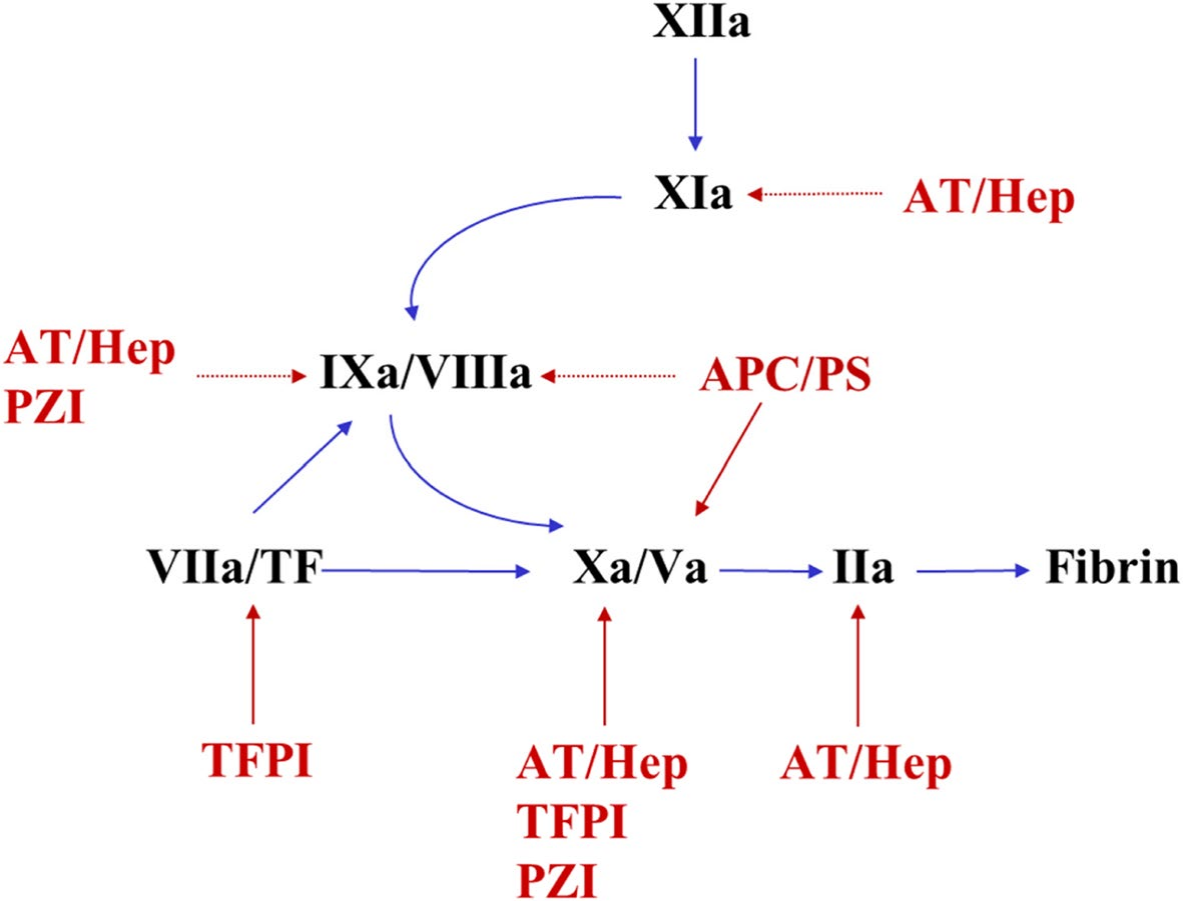
Regulation of blood coagulation. Antithrombin, tissue factor pathway inhibitor (TFPI), and activated protein C (APC) play major roles in the regulation of blood coagulation reactions. Antithrombin inhibits thrombin, FXa, FIXa, FXIa, and FXIIa when stimulated by physiologic heparan sulfate or pharmacologic heparins. TFPI neutralizes proteases of the extrinsic coagulation pathway, FVIIa, and FXa. APC exerts multiple protective homeostatic actions, including proteolytic inactivation of cofactors, Va and VIIIa, as well as direct cell-signalling activities involving protease-activated receptors (PAR) 1 and 3 and endothelial protein C receptor (EPCR). Protein Z-dependent protease inhibitor (PZI) inhibits FXa and XIa. The PZI-dependent inhibition of factor Xa is enhanced in the presence of protein Z

Various regulatory mechanisms control coagulation pathways to avoid excessive thrombus formation. The multistep mechanism of the coagulation cascade allows for more efficient control of the process. Antithrombin, tissue factor pathway inhibitors (TFPI), and protein C play major roles in blood coagulation regulation. The major anticoagulants and their targets are depicted in Fig. 2.

### Antithrombin

Antithrombin, formerly known as antithrombin III, is a major plasma glycoprotein of the serpin superfamily that regulates the proteolytic activity of procoagulant proteases of the coagulation system. The primary targets of antithrombin are thrombin, FXa, and FIXa. It can also inhibit FXIa, FXIIa, and TF-FVIIa [16].

Two important structural features that participate in the regulatory function of antithrombin include a reactive center loop that binds to the active site of coagulation proteases, trapping them in the form of inactive covalent complexes and a basic D-helix that binds to therapeutic heparins and heparan sulfate proteoglycans on vascular endothelial cells. The binding of the D-helix of antithrombin by therapeutic heparins promotes the reactivity of serpin with coagulation proteases via both conformational activation of serpin and a template (bridging) mechanism [16].

Conformational activation occurs when both FIX and FX are inhibited. The binding of a specific pentasaccharide sequence found in all forms of heparin, including low-molecular-weight and unfractionated heparin, results in a conformational change in the reactive center of antithrombin. This allows for enhanced access to the target protease and is known as allosteric activation of antithrombin [6].

The template mechanism describes the heparin-accelerated inhibition of thrombin, which involves bridging of antithrombin to the protease. This allows the two molecules to align, thereby enhancing the rate of complex formation. This mechanism requires longer-sized heparin molecules such as unfractionated heparin, amplifying the function of antithrombin by approximately 1,000 times [6].

### Tissue factor pathway inhibitor (TFPI)

TFPI is the major physiological regulator of TF-induced blood coagulation. TFPI inhibits the TF-FVIIa complex in an FXa-dependent manner, helping to control thrombin generation and ultimately fibrin formation [17, 18].

There are two major isoforms of TFPI in vivo, TFPIα and TFPIβ. TFPIα contains three Kunitz-type inhibitory domains (designated K1, K2, and K3) and is secreted by endothelial cells and platelets. Protein S is required to enhance its anticoagulant activity. Endothelial cells express TFPIα and TFPIβ that lack the third Kunitz domain, but platelets only produce TFPIα [17, 18].

After the initial generation of FXa in TF-bearing cells, subsequent generation of FXa is shut down by TFPI, which reacts with FXa to inactivate the TF-FVIIa-initiated coagulation cascade. TFPI binds to the active site of FXa through its second Kunitz domain, thereby inhibiting its proteolytic activity of FXa. Protein S, a cofactor, significantly accelerates this reaction by interacting with the third Kunitz domain of TFPI. TFPI inhibits the catalytic activity of TF-VIIa by forming a quaternary TF-FVIIa-FXa-TFPI complex. TFPIα also inhibits thrombin generation by prothrombinase during the initiation phase of coagulation, but not during the propagation phase. This is due to the structural differences of FXa- and thrombin-activated FVa [17, 18].

### Protein C

When bound to thrombomodulin (TM) on endothelial cells, thrombin converts protein C, a vitamin K-dependent plasma protein zymogen, into activated protein C (APC), which reduces thrombin production by inactivating FVa and FVIIIa. The anticoagulant activity of APC, which downregulates thrombin generation, is promoted by the cofactor protein S, which is also a vitamin K-dependent protein. Genetic deficiencies in proteins C and S are linked to the risk of venous thrombosis or neonatal purpura fulminans [19, 20].

The endothelial cell receptor TM and endothelial protein C receptor (EPCR) are required for efficient activation of protein C by thrombin. EPCR-bound APC has multiple direct cellular activities, whereas the dissociation of APC from EPCR allows the expression of the anticoagulant activity of APC. The beneficial cytoprotective and anti-inflammatory activities of APC require the cellular receptors protease-activated receptor-1 (PAR-1) and EPCR [19, 20].

### Protein Z-dependent protease inhibitor (PZI)

Protein Z-dependent protease inhibitor (PZI) is a serine protease inhibitor that inhibits FXa and XIa. The PZI-dependent inhibition of factor Xa is enhanced in the presence of protein Z, a vitamin K-dependent glycoprotein. In normal plasma, PZI is present in molar excess of protein Z, and all protein Z circulates in complex with PZI [21].

## Cross-talk of coagulation pathways with other host defence mechanisms

Recent investigations indicate that blood coagulation and thrombus formation may play major physiological roles in immune defense mechanisms in the human body. Blood coagulation and the immune systems of higher organisms are thought to have a common ancestral origin, as suggested by an analysis of the molecular evolution of their constituents. They play a dual role in the destruction of invading bacteria and prevention of body fluid loss. Although the fibrinolytic system is closely related to the coagulation system, it is also closely associated with digestive tract proteases on a molecular basis [22].

### The contact activation system

While no or only a minor role of the contact activation system has been implicated in hemostasis, epidemiological data with inherited factor deficiencies and studies in animals have demonstrated that the contact activation pathway might contribute to thrombosis. New anticoagulants targeting the components of the contact activation pathway, such as FXI and FXII, are being developed with the ultimate goal of preventing thrombosis without impairing hemostasis.

The contact activation system is central to the crosstalk with multiple physiological defense systems, including the coagulation, inflammation, fibrinolysis, and complement systems. Kallikrein, formed by FXIIa activation of prekallikrein, releases bradykinin from high-molecular-weight kininogens, resulting in inflammation. FXII activation can also lead to increased plasmin activation, which breaks down fibrin clots. FXIIa activates C1r and C1s via the complement pathway. Although the C1 inhibitor (C1INH) plays an important role in complement inhibition, it is also the main FXIIa inhibitor in vivo [15].

### Tissue factor (TF)

The discovery that TF circulates in the blood in both microparticle-bound and soluble forms has prompted further research on the role of TF beyond initiation of the tissue factor pathway. TF is expressed by many cell types, including T lymphocytes and platelets, in various pathological conditions such as acute and chronic inflammation and cancer. It has been suggested that TF may be a true surface receptor involved in intracellular signaling, cell survival, gene and protein expression, proliferation, angiogenesis, and tumor metastasis [23].

Cellular pathways that contribute to the non-coagulant roles of TF in thromboinflammatory diseases involve the signalling of distinct TF complexes with associated proteases through the PAR family of G protein-coupled receptors. Additional coreceptors, including EPCR and integrins, also play roles in directing subcellular localization and trafficking [24].

### Neutrophil extracellular trap (NET)

Neutrophils release neutrophil extracellular traps (NETs) that capture and kill bacteria and other pathogens, preventing their spread. Activated complement proteins stimulate NET formation, and NETs serve as a platform for complement activation. NETs can also function as scaffolds for thrombus formation during coagulation [25].

Activated coagulation factors in turn activate the complement system, alerting the immune system to recruit neutrophils. NET structures released by neutrophils can serve as direct scaffolds for thrombogenesis and complement activation [25].

## Conclusion

In conclusion, with the actively expanding field of hematology, it is important to have a strong understanding of the basics. Over the years, the intricate mechanism of coagulation has been revealed, deepening our understanding of this dynamic process. The blood coagulation cascade consists of 1) initiation by TF, 2) amplification by the intrinsic tenase complex, and 3) propagation on the activated platelets. The major regulators of blood coagulation include antithrombin, TFPI, and protein C. Recent studies have suggested that blood coagulation also plays an essential role in immune defense mechanisms. In the future, continuous research is warranted to further understand and develop foundational concepts in hematology.

## Data Availability

No datasets were generated or analysed during the current study.

## Abbreviations

AT: Antithrombin
Hep: Heparin
TFPI: Tissue factor pathway inhibitor
APC: Activated protein C
PS: Protein S
TM: Thrombomodulin
PZI: Protein Z-dependent protease inhibitor
